# Supplementary material for: Wnt-Dependent Activation of ERK Mediates Repression of Chondrocyte Fate during Calvarial Development
Source: J Dev Biol. 2021 Jun 27;9(3):23. doi: 10.3390/jdb9030023 (PMC8293402; doi:10.3390/jdb9030023)
Supplement: Supplementary file 1 [file jdb-09-00023-s001.zip › Final Supplement_PDF/Final_Supplement2.pdf]

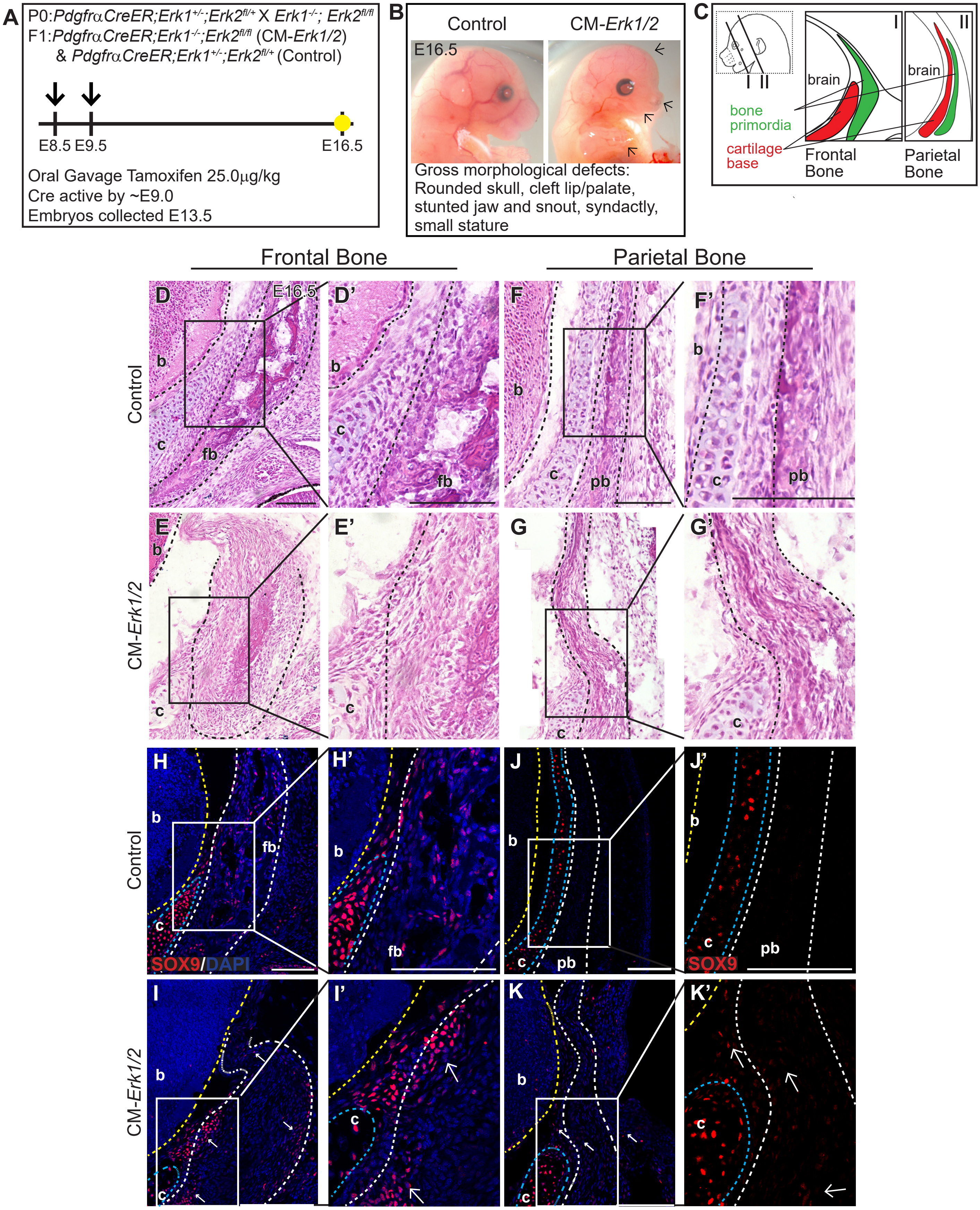

Figure S2. Loss of ERK1/2 in the cranial mesenchyme results in diminished calvarial bone and ectopic expression of SOX9 at E16.5.

(A) Schematic depicting the gavage regimen and timing of deletion of *Erk2*. (B) Whole mount photos depicting gross morphological defects. (C) Schematic indicating the orientation and plane of specimens. (D-G') H&E of CM-*ERK1/2* mutants and controls at E16.5. (H-K') Immunofluorescence of cartilage marker SOX9. Arrows denote ectopic SOX9 expression in bone region, arrowheads denote diminished cartilage base of mutant. Presumptive fb, frontal bone, and pb, parietal bone region is outlined by white dashed lines, yellow dashed line demarcates b, brain, blue dashed line outlines c, cartilage base. n = 1
