# Supplementary material for: Wnt-Dependent Activation of ERK Mediates Repression of Chondrocyte Fate during Calvarial Development
Source: J Dev Biol. 2021 Jun 27;9(3):23. doi: 10.3390/jdb9030023 (PMC8293402; doi:10.3390/jdb9030023)
Supplement: Supplementary file 1 [file jdb-09-00023-s001.zip › Final Supplement_PDF/Final_Supplement4-Wls DDPCR data.pdf]

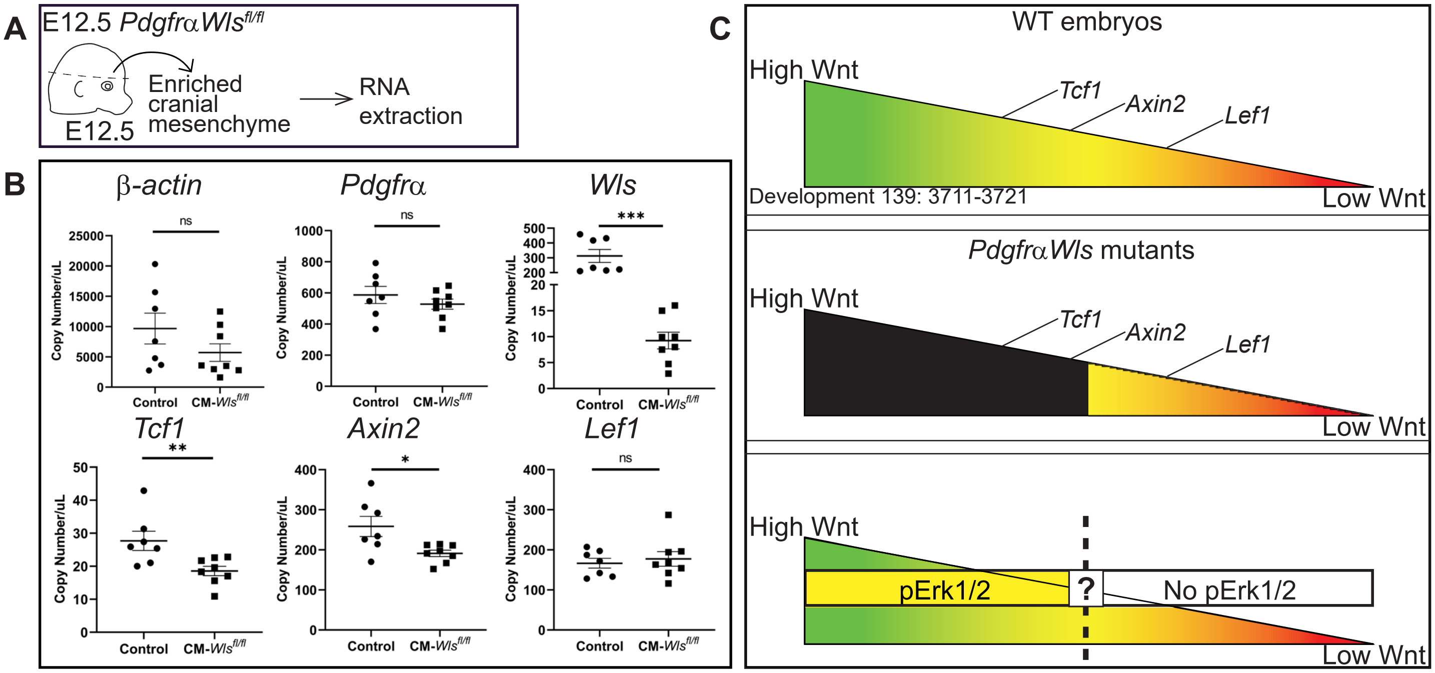

Figure S4. Mesenchyme *Wntless (Wls)* is required for variable expression of Wnt target genes. (A) Schematic depicting work flow and tissue source for ddPCR analysis in panel B. (B) Total copy number/μL of indicated genes as determined by DDPCR. (C) Hypothesized model of Wnt signaling threshold within the cranial mesenchyme of Wild-type (top), mesenchyme *Wls* mutants (middle) and putatitve model for Wnt-dependent Erk activation (bottom).
