# Supplementary material for: Wnt-Dependent Activation of ERK Mediates Repression of Chondrocyte Fate during Calvarial Development
Source: J Dev Biol. 2021 Jun 27;9(3):23. doi: 10.3390/jdb9030023 (PMC8293402; doi:10.3390/jdb9030023)
Supplement: Supplementary file 1 [file jdb-09-00023-s001.zip › Final Supplement_PDF/Final_Supplemental.1_Erk qPCR data.pdf]

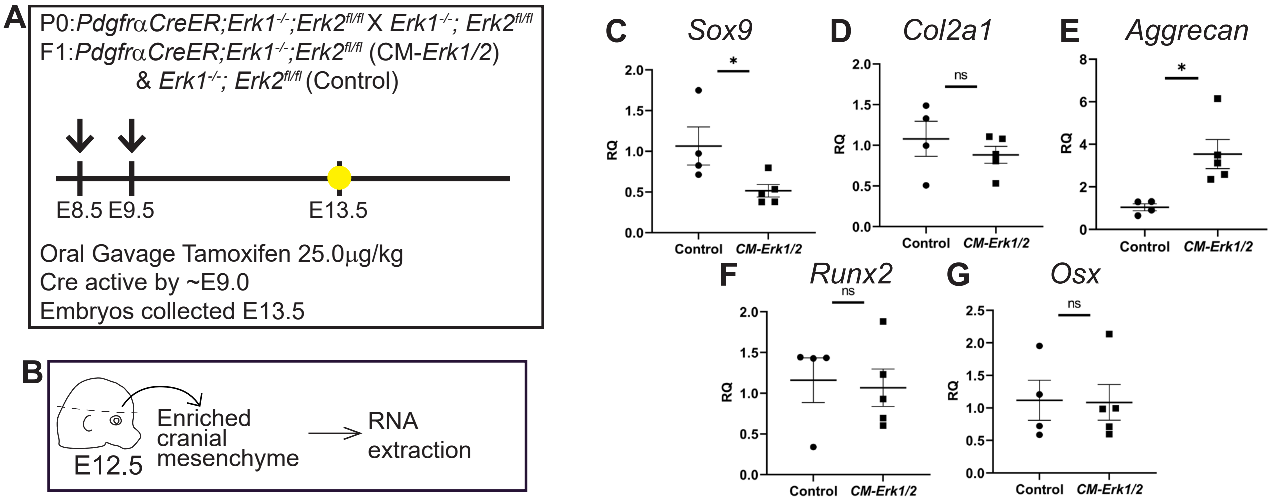

Figure S1. RT-qPCR of cartilage and bone lineage markers in CM-ERK1/2 and controls. Schematics depicting the gavage regimen and timing of deletion of *Erk2* (A), and the work flow and tissue source for the RNA analysis (B). RT-qPCR analysis of cartilage markers *Sox9* (C), *Col2a1* (D), and *Aggrecan* (E) and bone markers *Runx2* (F) and *Osx* (G). *n* = 4-5
