# Supplementary material for: Wnt-Dependent Activation of ERK Mediates Repression of Chondrocyte Fate during Calvarial Development
Source: J Dev Biol. 2021 Jun 27;9(3):23. doi: 10.3390/jdb9030023 (PMC8293402; doi:10.3390/jdb9030023)
Supplement: Supplementary file 1 [file jdb-09-00023-s001.zip › Final Supplement_PDF/Final_Supplemental3.pdf]

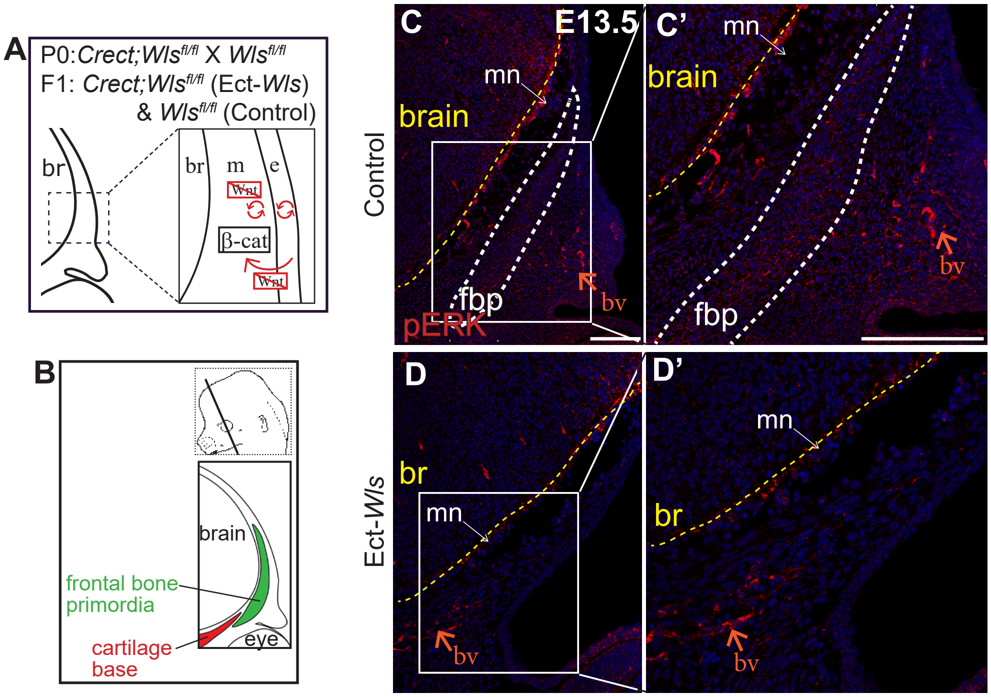

Figure S3. Ectoderm *Wntless (Wls)* mutants lose ERK activation in cranial mesenchyme.
(A) Schematic depicting the status of Wnt signaling in the cranial mesenchyme and overlying ectoderm in this mouse model of Wnt signaling deletion. β-catenin remains intact within the cranial mesenchyme. The ectoderm is unable to secrete Wnt ligands or undergo Wnt signaling. Wnt signaling is lost in the mesenchyme. (B) Schematic indicating the plane and orientation of specimen. (C-D') Immunoflourescence of pERK1/2 showing loss of ERK1/2 activation in *Creect;Wls*$^{fl/+}$ (control) and *Creect;Wls*$^{fl/fl}$ (Ect-*Wls*). n=2 biological replicates. Yellow lines demarcate the brain, white lines border the presumed frontal bone primordia (fbp). White arrows identify pERK signal within the frontal bone primordia, orange arrow points to pERK signal within the vasculature; bv, blood vessel(s)
